# Serial passage of HIV-2F: a pigtail macaque model for HIV emergence

**DOI:** 10.1186/1471-2334-14-S2-P57

**Published:** 2014-05-23

**Authors:** Nell G Bond, Stephanie L Feely, Christopher Monjure, Michael Lauck, David O’Connor, Nick Manness, Preston A Marx

**Affiliations:** 1 Tulane University School of Public Health and Tropical Medicine, New Orleans, Louisiana, USA; 2 Tulane National Primate Research Center, Covington, Louisiana, USA; 3 University of Wisconsin, Madison, Wisconsin, USA

## 

The AIDS pandemic affects over 35 million people worldwide. Human immunodeficiency types 1 and 2 (HIV-1 and HIV-2), the etiologic agents of AIDS, originated from simian immunodeficiency viruses of chimpanzees (SIVcpz) in Cameroon and SIVsm from sooty mangabeys in West Africa. While the simian origin of HIV is well established, how the virus adapted to humans is poorly understood. The bulk of HIV-2 morbidity and mortality is caused by a few strains; however, new pathogenic subtypes of HIV-2 continue to emerge (HIV-2F, 2008 and HIV-2H, 2004) underscoring the need for deeper understanding of the mechanisms behind the adaption of these viruses to humans. This study aims to test the serial passage theory of HIV emergence and elucidate adaptive mechanisms to a new host using the newly emerged, pathogenic HIV-2F virus in an in vivo pigtail macaque (PTM) model.

We inoculated one pigtail macaque with HIV-2F isolated from the patient followed by serial passage into 2 PTMs. Blood, lymph node; endoscopy and vaginal wash were collected. An HIV-2F specific quantitative PCR (qPCR) assay was developed (LOQ=1.9 log VC/mL) for this experiment.

All three PTMs were infected; reaching peak PVLs between 6.0 and 7.2 log viral copies/milliliter. PTM KF25, passage one, cleared the virus by day 42 post inoculation (PI) and remains qPCR negative at day 344 PI. KF26 and KF24, passages two and three, cleared the virus following acute infection by days 42 and 60 respectively. Importantly, virus rebounded in the 2nd and 3rd passage animals at day 150 and 120, respectively, and remains between 5.6 and 3.2 log VC/mL.

Following one passage in PTMs, HIV-2 F escaped host control and sustained replication, showing that the virus adapted to the new host via serial passage. Sequence analysis comparing peak and re-emergent virus is ongoing and will be completed for presentation. Flow cytometry analysis to correlate changes in VL with cytokine markers of pathogenesis is in process. Clinical monitoring is ongoing to assess pathogenesis of passaged HIV-2F.

